# Multi-omic Characterization of Intraspecies Variation in Laboratory and Natural Environments

**DOI:** 10.1128/mSystems.00764-21

**Published:** 2021-08-24

**Authors:** Megan G. Behringer

**Affiliations:** a Department of Biological Sciences, Vanderbilt University, Nashville, Tennessee, USA; b Department of Pathology, Microbiology, and Immunology, Vanderbilt University Medical Center, Nashville, Tennessee, USA; c Vanderbilt Microbiome Initiative, Nashville, Tennessee, USA

**Keywords:** ecotypic variation, experimental evolution, population genomics, systems biology, metagenomics, systems microbiology

## Abstract

Investigation of microbial communities has led to many advances in our understanding of ecosystem function, whether that ecosystem is a subglacial lake or the human gut. Within these communities, much emphasis has been placed on interspecific variation and between-species relationships. However, with current advances in sequencing technology resulting in both the reduction in sequencing costs and the rise of shotgun metagenomic sequencing, the importance of intraspecific variation and within-species relationships is becoming realized. Our group conducts multi-omic analyses to understand how spatial structure and resource availability influence diversification within a species and the potential for long-term coexistence of multiple ecotypes within a microbial community. Here, we present examples of ecotypic variation observed in the lab and in the wild, current challenges faced when investigating intraspecies diversity, and future developments that we expect to define the field over the next 5 years.

## COMMENTARY

Estimated to account for almost five million species, prokaryotes are the most diverse and abundant organisms on earth (1). Prior to the genomic era, microbiologists suspected this incredible diversity in microbial species, but the lack of a universal marker hindered the ability to effectively describe the vast spectrum of microbial life. Development of phylogenetic approaches based on the 16S ribosomal DNA loci by Carl Woese et al. not only resolved *Archaea* as a distinct domain of the prokaryotic tree of life but also provided a way to distinguish divergent prokaryotic taxa and assess evolutionary relatedness (2, 3). Yet, despite the descriptive capability of this universal locus, investigation of microbial communities was still largely culture dependent until the late 2000s, and a swath of diversity represented by “unculturable” bacteria remained elusive. In response, efforts to harness culture-independent techniques were made and protocols utilizing microarrays (4) and pyrosequencing (5) were subsequently developed. Ultimately, it was the pairing of 16S rRNA gene analysis with next-generation sequencing which resulted in a widely accessible, low-cost, and high-throughput technique for the broad quantification of microbial diversity. In the 10 years since, 16S amplicon sequencing has fueled thousands of microbiome studies (6). These investigations have been vital to describing the tremendous role of microbes in maintaining ecosystem function and suggest that the composition of microbial communities can act as an indicator of host or environmental ecosystem health (7–9). While 16S sequencing has been powerful for resolving microbial diversity at the level of genera and species, considerable advances in computational power and sequencing technology have further revealed a wealth of intraspecies genetic variation through shotgun metagenomic sequencing. As such, the functional diversity of microbial communities may extend beyond the species level, and the presence of multiple strains or ecotypes within a microbial species population can also be functionally important (10).

Subpopulation structure and ecotypic diversification is a common evolutionary outcome in heterogeneous environments. In the lab, populations of Pseudomonas fluorescens and Escherichia coli were demonstrated to diversify spatially based on varying ability to form biofilms and preference for the surface-air interface niche (11, 12). When specifically selected to progress through regular biofilm cycles due to cultivation in batch culture with plastic beads, Burkholderia cenocepacia populations were observed to diversify into three ecotypes representing a network of cross-feeding interactions (13). Fixing the spatial location of E. coli cells by culture in microfluidic channels similarly resulted in the creation of metabolic microhabitats. Here, ecotypes arose based on cross-feeding between glucose-fermenting cells and cells that evolved to utilize acetate waste products as a source of essential carbon.

While the nature of heterogeneous environments likely encourages diversification due to the presence of structured niches, adaptive diversification is also observed in more homogeneous conditions. Richard Lenski’s Long-term Evolution Experiment (LTEE) bacteria, a collection of 12 E. coli populations that have been famously evolving for over 70,000 generations, have been cultivated in flasks. Despite the LTEE’s relatively simple and well-mixed culture environment, more than half of the populations have exhibited the evolution of long-term coexisting ecotypes (14, 15), including a population where diversification resulted in the novel ability to aerobically uptake and utilize citrate (16). Another simple environment supporting the evolution of ecotypes is continuous culture in chemostats, where again the emergence of glucose and acetate specialists was observed in E. coli populations (17, 18).

While it would be easy to dismiss these examples of diversification as a laboratory artifact and a result of monospecies populations cultivated in the absence of interspecific competitors, significant within-species variation is also found in clinical and environmental settings. For example, in cystic fibrosis lung-like conditions, Pseudomonas aeruginosa was reported to rapidly diversify (19), and long-term coexistence of ecotypes has been observed in clinical samples from a single host (20). Moreover, sampling of coastal waters revealed coexisting ecotypes of the bacterioplankton Vibrio splendidus that were likely based on resource partitioning (21), and *Curtobacterium* soil ecotypes exhibit specialization along a climate gradient (22). These examples illustrate that ecotypic variation is likely widespread across environmental contexts. Thus, there is a need for more in-depth investigation of how microbes adapt and how different genetic backgrounds or culture conditions permit/inhibit ecotypic diversification.

Our group’s research is interested in how interspecies variation is generated and maintained in bacterial populations. These studies can range from investigations of spontaneous mutation rates using a mutation accumulation/whole-genome sequencing (MA/WGS) approach to experimental evolution studies examining how space and stress shape bacterial adaptation. Using a multi-omic approach that combined time-series metagenomic sequencing with genomic, transcriptomic, and exometabolomic analysis of isolated clones, we demonstrated that daily-transferred E. coli populations maintained in spatially complex (16- by 100-mm glass culture tubes) and nutrient-rich (LB-Miller broth) media rapidly diversify into ecotypes based on location (biofilm versus planktonic) and then utilization of amino acid resources (12). These ecotypes coexisted long-term (>10,000 generations) and were a specific adaptive outcome of culture under our heterogeneous conditions, as their evolved fitness benefits did not translate to more-homogeneous environments.

Going forward, our group will focus more on how environment influences the evolution of ecotypes and if any genetic backgrounds are particularly permissive/inhibitory to the maintenance of subpopulation structure (23). Beyond lab-based studies, we also intend to apply our understanding of adaptive diversification and bacterial population genetics to clinical investigations. As has been established, if bacteria are given the niche availability, then they can diversify and inhabit novel spaces. This potential to evolve subpopulations also represents the ability to maintain multiple genetic backgrounds on which antimicrobial resistance can emerge. Thus, understanding how pathogen populations evolve throughout the course of an infection is of major interest to us, especially in recurrent contexts. To this end, we are partnering with physicians, clinical microbiologists, and microbiologists specializing in pathogenesis to gain a deeper understanding of microbial population dynamics and their effect on disease.

## CHALLENGES AND FUTURE OF THE FIELD

To date, the identification of ecotypes and the characterization of their interactions remain challenging. On the genomic level, short reads produced by next-generation sequencing platforms provide the accuracy to confidently identify polymorphisms within a bacterial population but fall short in the ability to phase these polymorphisms across sequencing reads to determine which mutations are present within an individual. To overcome this issue, computational approaches have been developed to assist in the identification of ecotypes, such as clonal reconstruction (a clone-informed maximum-likelihood method to parsing time-series metagenomic sequencing of populations [24]) or clade-aware HMM (a hidden Markov model that infers if a mutation is fixed or polymorphic within a basal, major, or minor clade from time-series sequencing [15]). While these methods have been proven powerful for resolving subpopulation structure when applied to experimental evolution studies, they are not without their shortcomings: clonal reconstruction relies on additional sequencing of clonal isolates, which is problematic for unculturable taxa, clade-aware HMM struggles with more complex population structures, and both are computationally challenging. Future development of these and other computational approaches implementing machine learning, as well as current progress increasing the accuracy of single-bacterial-cell DNA sequencing (25) and single-molecule sequencing platforms, will increase the accessibility of studying the diversity within microbial populations in a broad variety of contexts.

Once ecotypes are identified, characterizing their role within the population or microbiome has additional complications. Microbes often exhibit emergent behavior, and their phenotypic expression may significantly vary in isolation from how they respond in the presence of their community (26). On a broader scale, this behavior can be assessed with metatranscriptomics. However, due to challenges introduced by genetic relatedness, assessing ecotype-specific differences in global gene expression from coculture is impossible, since most sequencing reads will lack any identifying information that could otherwise indicate from which ecotype that transcript may have originated. Fortunately, current developments in bacterial single-cell RNA sequencing methods (27–29) which bypass the complications of extremely low RNA yields from prokaryotic cells and nonpolyadenylated mRNAs will heavily mitigate this issue in the future. These methods will not only permit the characterization of ecotypes but also distinguish differential gene expression for closely related species in coculture and investigations of phenotypic heterogeneity (30). Lastly, without time-series sampling, true subpopulation structure cannot be distinguished from transient polymorphism on its way to fixation or extinction. As such, biobanking of microbiome samples will become increasingly important to investigate within-species variation in clinical or environmental contexts. Connecting the diversity within these communities to the presence of pathogenic or ecologically important isolates will empower microbiologists to identify microbiomes in transition between healthy and disease states so that future interventions may be developed.
